# Medical Education

**Published:** 1900-11-17

**Authors:** 


### The Hospital

A Journal of

The Medical Sciences and Hospital Administration.

Vol. XXIX.—No. 738.] Edited by Sir Henry Burdett, K.C.B., and Solomon C. Smith, M.D., M.R.C.P. [November 17, 1900.

### Medical Education.

The remarks made last week by Sir Michael Foster, M.P., in distributing prizes to the students of St. George's Hospital do not stand alone as indications that the whole system and methods of medical education are in need of complete revision, if they are to supply a profession fully equipped with the aids to healing which modern science has afforded, and which it seems likely to continue affording in an increasing ratio. Sir Michael complained that the great opportunities offered by our hospitals for the advance of medical knowledge were not adequately laid hold of; and he quoted what he declared to be the general opinion of American teachers, to the effect that, when they sent their graduates to England, although these graduates were impressed with what they saw as to the unsurpassed excellence with which the London schools trained the medical student, they themselves, as graduates seeking post-graduate aid, got very little from the general hospitals, and had to go away from England, often against their wish, in order to pursue research abroad. The remedy which Sir Michael suggested was to have a properly-equipped clinical laboratory attached to every hospital, and regarded as a necessary charge upon its funds, as much as drugs or surgical appliances. The object of such a laboratory would be to carry out a complete system of chemical, physical, biological, bacteriological, and other investigations into the causes and interpretation of the symptoms presented by patients. At the same hospital, a few weeks earlier, in his introductory lecture, Dr. Penrose told the students that no single subject would be of greater importance to them than that of physiological chemistry. He went on to express the wish that every medical student, at some period of his training, should spend a sufficient time in the chemical laboratory to become as familiar with the fundamental principles of the subject as he must be with human anatomy and with the physics of physiology. A large part of the medical science of the future will manifestly be the direct outcome of what is called physiological or organic chemistry, and between organic and inorganic chemistry it is nowhere possible to draw a dividing line. Even to-day there are maladies which a practitioner who was an accomplished chemist would treat upon a basis of complete clinical examination and of assured knowledge, but which a practitioner who was not an accomplished chemist could only treat by the rule of thumb application of precepts which have been laid down by others, and which he tried to remember by rote. Such a practitioner, regarded as a physician, is much in the same case as that of a surgeon ignorant of anatomy who was constrained to amputate by reason of his inability to dissect. Anatomy is taught, and is made the subject of examinations which are, perhaps, sufficiently stringent; but where and to what extent is chemistry taught to medical students, and where and to what extent are they examined in it? We referred last week to the recalcitrancy of the two Royal Colleges of London with regard to the very moderate requirements of the General Medical Council; and we cannot but recognise, in this recalcitrancy, a tendency to endeavour to stem the inevitable progress of events. It is daily becoming more clear that the old standards and methods of medical education must change, and give place unto the new; and it is in the power of the nineteen licensing bodies with which Great Britain is blessed, and upon whom alone the Legislature, in the humorous phrase of Sydney Smith, has conferred the power of suspending the sixth commandment, either to place themselves at the head of a movement for this purpose, or to make futile efforts to withstand it. If the chemists and physiologists of the next generation should be compelled to say, of the young medical practitioners commencing life around them, "These men have not been so educated as even to be able to understand the full bearings of our work," the day will not be far distant on which the licensing bodies will be called upon to give an account of their stewardship, and will be in considerable danger of finding themselves and their curricula superseded by the State. They have now to learn that rights are commensurate with duties; and that even vested interests, of which, as the equivalents of property, the Legislature is bound to be scrupulously careful, cannot permanently be maintained if the purposes for which those interests were created are suffered to remain unfulfilled. The difficulty probably arises, in great part, from the natural tendency of corporations to be governed by their senior members, whose power of adjusting themselves to a changing environment is already in course of diminution. If the Committee of Management of the Conjoint Board of England were reinforced by a few of the leaders of investigation, it is not improbable that the policy which has hitherto prevailed at Examination Hall might be modified, and modified with advantage alike to the profession and to the public.

